# Genotypic differences in architectural and physiological responses to water restriction in rose bush

**DOI:** 10.3389/fpls.2015.00355

**Published:** 2015-05-26

**Authors:** Camille Li-Marchetti, Camille Le Bras, Daniel Relion, Sylvie Citerne, Lydie Huché-Thélier, Soulaiman Sakr, Philippe Morel, Laurent Crespel

**Affiliations:** ^1^ASTREDHOR – Institut Technique de l’Horticulture, ParisFrance; ^2^Agrocampus Ouest, Unité Mixte de Recherche 1345, Institut de Recherche en Horticulture et Semences (INRA-Agrocampus Ouest-Université d’Angers), AngersFrance; ^3^INRA, Institut Jean-Pierre Bourgin Centre de Versailles-Grignon, Unité Mixte de Recherche 1318 (INRA-Agro-ParisTech-CNRS), VersaillesFrance; ^4^INRA, Unité Mixte de Recherche 1345, Institut de Recherche en Horticulture et Semences, (INRA-Agrocampus Ouest-Université d’Angers), BeaucouzéFrance

**Keywords:** architectural analysis, genotype × environment interaction, *Rosa*, water restriction tolerance, hormones

## Abstract

The shape and, therefore, the architecture of the plant are dependent on genetic and environmental factors such as water supply. The architecture determines the visual quality, a key criterion underlying the decision to purchase an ornamental potted plant. The aim of this study was to analyze genotypic responses of eight rose bush cultivars to alternation of water restriction and re-watering periods, with soil water potential of -20 and -10 kPa respectively. Responses were evaluated at the architectural level through 3D digitalization using six architectural variables and at the physiological level by measuring stomatal conductance, water content, hormones [abscisic acid (ABA), auxin, cytokinins, jasmonic acid, and salicylic acid (SA)], sugars (sucrose, fructose, and glucose), and proline. Highly significant genotype and watering effects were revealed for all the architectural variables measured, as well as genotype × watering interaction, with three distinct genotypic architectural responses to water restriction – weak, moderate and strong – represented by Hw336, ‘Baipome’ and ‘The Fairy,’ respectively. The physiological analysis explained, at least in part, the more moderate architectural response of ‘Baipome’ compared to ‘The Fairy,’ but not that of Hw336 which is an interspecific hybrid. Such physiological responses in ‘Baipome’ could be related to: (i) the maintenance of the stimulation of budbreak and photosynthetic activity during water restriction periods due to a higher concentration in conjugated cytokinins (cCK) and to a lower concentration in SA; (ii) a better resumption of budbreak during the re-watering periods due to a lower concentration in ABA during this period. When associated with the six architectural descriptors, cCK, SA and ABA, which explained the genotypic differences in this study, could be used as selection criteria for breeding programs aimed at improving plant shape and tolerance to water restriction.

## Introduction

The shape of a plant and, therefore, its architecture determine its visual quality, a key criterion underlying the decision to purchase an ornamental potted plant. It is also a key factor in terms of yield, i.e., the number of floral stems for a cut flower (Kool, 1996). Plant architecture is the result of the positioning of aerial organs in space according to organization rules specific to each species. It depends on two processes, growth and branching, which are controlled by genetic and environmental factors, including the quantity and quality of light (Kawamura and Takeda, 2002; Niinemets and Lukjanova, 2003; Evers et al., 2006; Girault et al., 2008; Rameau et al., 2015), temperature (Khayat and Zieslin, 1982; Battey, 2000) as well as water supply (Cameron et al., 2006; Burnett and van Iersel, 2008; Demotes-Mainard et al., 2013; Huché-Thélier et al., 2013). Climate forecast models consider an average global rise in temperature of ∼2∘C between now and 2100, accompanied by an increase in the frequency of heat waves and droughts (IPCC, fifth assessment, 2013).

There are many plant responses to water deficit (Chaves and Oliveira, 2004; Kooyers, 2015). They can be characterized by morphological and therefore, architectural modifications which could be explained by physiological adjustments. At the morphological level, water deficit can lead to a reallocation of the biomass and, therefore, a modification of the size of different organs, with an inhibition of stem growth, inhibition of root growth, and a decrease in the leaf surface (Chaves and Oliveira, 2004; Jaleel et al., 2008; Niu and Rodriguez, 2009). At the physiological level, these responses are mediated, among other things, by changes in hormonal balances (Peleg and Blumwald, 2011). Therefore, an increase in the concentration of abscisic acid (ABA) is observed, leading to stomatal closure to reduce water losses in the plant due to transpiration (Wilkinson and Davies, 2002; Acharya and Assmann, 2009). In addition to ABA, other hormones are also involved in the plant’s responses to water deficit, including cytokinins (CKs; Ha et al., 2012), salicylic acid (SA; Horvath et al., 2007), and jasmonic acid (JA; Brossa et al., 2011). Responses to water deficit may also give rise to the synthesis of osmoprotectors such as proline (Delauney and Verma, 1993; Maggio et al., 2002), osmoregulators such as soluble sugars (Watanabe et al., 2000) and photosynthetic adjustments (Flexas and Medrano, 2002). Responses within the same species can vary from one genotype to another, as has already been shown for cereal species: wheat (Moinuddin et al., 2005), barley (Rizza et al., 2004), and soybean (Hufstetler et al., 2007). More recently, Granda et al. (2014) revealed two responses among nine clones (i.e., cultivars in horticulture) of *Eucalyptus globulus* submitted to water stress. One was characterized by the implementation of mechanisms aimed at maintaining a high water content, accompanied by a reduction in plant growth, whereas the other was characterized by a reduced water content, osmotic adjustments and an increase in growth.

Few studies have focused on the effect of a water deficit on plant architecture. For example, to assess the effect of a water deficit on plant architecture, the length and/or the diameter of the stems were measured for *E. globulus, Pinus sylvestris,* and *Catharanthus roseus* (Jaleel et al., 2008; Pearson et al., 2013; Granda et al., 2014), and the number of metamers, the number and length of stems were measured for *Cotinus coggygria* and *Forsythia* x *intermedia* (Cameron et al., 2006, 2008).

**FIGURE 1:**
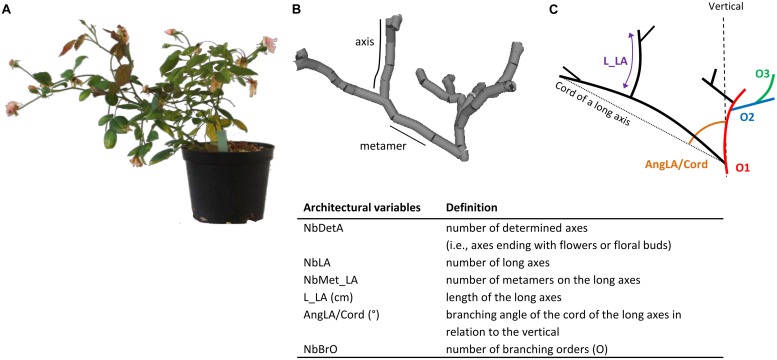
**Plant photography before phenotyping by 3D digitalization **(A)**; plant architecture with two components (metamer and axis; **B**); simplified representation with three branching orders: Order 1 (O1), Order 2 (O2), and Order 3 (O3; **C**)**.

The phenotyping of plant architecture is long and laborious. It can be broken into architectural components: axes and metamers, where a metamer is the unit formed by an internode, a node, its axillary bud and a leaf (White, 1979). These components can be described by architectural variables: morphologically (length, diameter, etc.), topologically (order of branching, etc.), and geometrically (branching angle, etc.; Godin, 1999). This architectural analysis was first applied to trees such as walnut (Barthélémy et al., 1995) and birch (Caraglio, 1996), and more recently to rose by Morel et al. (2009) and Crespel et al. (2013). According to Crespel et al. (2013), six variables were necessary and sufficient to describe the architecture and its variation in rose (**Figure 1**).

Available studies on the effect of water deficit on rose are marked by a limited number of genotypes (ranging from 1 to 4 depending on study), phenotypic variables (dry weight of stems, leaves and roots, number of flowers and buds, flower development), and physiological variables (water content, gas exchanges, chlorophyll fluorescence, carbohydrate content, ion content, proline content, malondialdehyde content, superoxide dismutase, and ascorbate peroxidase activities; Williams et al., 2000; Jin et al., 2006; Bolla et al., 2009; Niu and Rodriguez, 2009; Cai et al., 2012). Different responses were observed between genotypes. Nevertheless, no measurement was made to assess the effect of water deficit on plant architecture.

The aim of our study was to assess genotypic responses to the application of an alternation of water restriction periods and re-watering periods on the architecture of plants aged from 5 to 6 months, for eight rose genotypes with contrasted shapes. The architecture of the plants was characterized on the basis of six variables defined by Crespel et al. (2013). In order to explain the architectural differences revealed between genotypes, a physiological analysis that included the measurement of stomatal conductance, water content and contents in hormones, sugars, and proline was carried out.

Improving our knowledge of genotypic responses to water deficit is a first step toward a better characterization of genitors for breeding programs, aiming to the creation of cultivars that are tolerant to water restriction without significant effect on plant shape.

## Materials and Methods

### Plant Material

Five bush roses cultivars with contrasting shapes ranging from spreading to upright were initially chosen: ‘Baipome’ Pink Gnome, ‘Blush Noisette,’ ‘Old Blush,’ ‘Perle d’Or,’ and ‘The Fairy,’ in addition to three hybrids with intermediate shapes, Hw20, Hw154, and Hw336. The latter are derived from a cross between a dihaploid of *Rosa hybrida* L. H190 and a hybrid of *R. wichurana* Crép. (Crespel et al., 2002).

### Experimental Conditions

Plants were obtained from cuttings of 10-month-old plants grown in pots in the experimental facilities of the IRHS (French Research Institute on Horticulture and Seeds) in Angers. Cuttings consisted of a single metamer from the median zone of the stems. Cuttings were taken in January and planted in plugs (diameter: 35 mm; height: 40 mm) composed of a non-woven fabric containing a mixture of fine peat and perlite. They were placed under a plastic tunnel in a greenhouse for rooting. The average temperature was 18∘C at night and 22∘C during the day, and the relative humidity was maintained at saturation by a fine mist humidifier. After 5 weeks, the plants were planted in 0.5-L pots and then in 2-L pots a month later. The experiment was conducted in a greenhouse on 4.5-m^3^ benches equipped with a nutrient solution tank, with one cultivar per bench and 60 plants per cultivar. The soil water potential was measured by a tensiometer and sub-irrigation was triggered when a defined threshold was reached. Mineral nutrition was provided by fertilization with a liquid fertilizer (N-P2O5-K2O, 3:2:6, pH = 6.5, electro-conductivity = 1.2 ms.cm^-1^). Minimum air temperature was maintained at 18∘C, with aeration at 20∘C. Relative humidity was maintained at 70%. No complementary lighting was applied. For each cultivar, plants were randomly divided into two batches: a control treatment, characterized by water comfort, and a water restriction (WR) treatment, each one composed of 30 plants.

### Water Restriction Experiment

**FIGURE 2:**
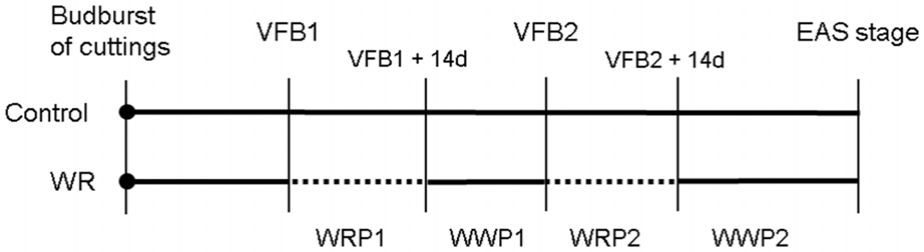
**Application of the water restriction (WR) treatment based on developmental stage of the primary axis [bud burst of the rooted cutting, visible flower bud of the order 1 axis (VFB1), visible flower bud of the order 2 axes (VFB2) and elementary architectural structure (EAS) stage]**. 14-days (14d) periods of water restriction (WRP1 and WRP2) are indicated by dotted lines and periods of re-watering (WWP1 and WWP2) by continuous lines.

For the control treatment, the soil water potential was maintained at -10 kPa throughout the experiment. For the WR treatment, plants were subjected to two successive water restriction periods (WRPs) of 14 days each, WRP1 and WRP2, respectively (**Figure 2**). During these two WRPs, the soil water potential was maintained at -20 kPa by manual watering. WRP1 was applied when the floral bud of the order 1 axis became visible (VFB1), and WRP2 when the floral buds of the order 2 axes were visible (VFB2). Since the beginning of the WRP depends on the phenological stage, WRP were not necessarily synchronized between cultivars. After each WRP, plants were well-watered [corresponding to well-watered periods (WWPs)] at a soil water potential of -10 kPa, WWP1 and WWP2, respectively. For each treatment and cultivar, the soil water potential was measured by a tensiometer and sub-irrigation was triggered when a defined threshold was reached.

The soil water potential, alternation of WRP/WWP and the duration were chosen according to a previous study made by Demotes-Mainard et al. (2013) on rose.

### Architectural Measurements

Plant architecture is characterized by two architectural components, the axis and the metamer. Each component was described by architectural variables: morphologically (length, diameter, etc.), topologically (order of branching, etc.), and geometrically (branching angle, etc.). Two types of axes can be distinguished in roses, short axes that are composed of 1–4 metamers, and long axes that are composed of five metamers or more (Morel et al., 2009). The architectural analysis was carried out at the plant and axis scales, based on six architectural variables that previously proved to be sufficient to describe the plant architecture (Crespel et al., 2013): (i) at the plant scale, the number of determined axes (i.e., axes ending with flowers or floral buds; NbDetA) and the number of long axes (NbLA); (ii) at the axis scale, the number of metamers on the long axes (NbMet_LA), the length of the long axes (L_LA), the branching angle of the cord of the long axes in relation to the vertical axis (AngLA/Cord), and the number of branching orders (NbBrO; **Figure 1**).

Architectural measurements were performed at the elementary architectural structure (EAS) stage (Crespel et al., 2013) and took place from 21 May to 8 July 2013. Plants that had reached this stage were stored for no longer than 1 week at 2∘C in a dark cold chamber in order to prevent their growth and branching (Girault et al., 2008; Djennane et al., 2014) and avoid any experimental bias. Ten plants per cultivar for each treatment were randomly chosen and defoliated. Architectural measurements were carried out using a Fastrack digitizer (Polhemus, Colchester, VT, USA) and data were recorded using PiafDigit software (Donès et al., 2006). This software makes it possible to both store measurements made at the two observation scales and to construct a 3D real-time representation of the plant architecture. Data are coded in MTG (multi-scale tree graph) format (Godin and Caraglio, 1998). Variables were extracted from these data using AMAPmod software (Godin et al., 1999).

### Architectural Data Analysis

All the data were subjected to a two-way analysis of variance (ANOVA) to determine genotype and watering effects and their interaction. Multiple comparisons of means (Newman–Keuls test) were performed. Differences with *p* < 0.05 were considered to be significant. Data analyses were carried out using R 3.0.2 software. The percentages of the differences between WR-treated plants and the control for each architectural variable were calculated and summed as absolute values. Each value was then normalized by dividing by the value corresponding to the least responsive genotype, Hw336. The intensity of the architectural response to water restriction of each genotype was then assessed based on these ratios.

### Physiological Measurements

Among the eight genotypes used of architectural analysis, three cultivars were selected for the physiological characterization: ‘Baipome,’ Hw336, and ‘The Fairy’ since they were representative of the three different intensities of architectural responses observed in this study (see Results). Thirteen water stress-related variables were measured as described below.

### Stomatal Conductance (g_s_)

Stomatal conductance (g_s_) was measured *in situ* every 2 days from the onset of WRP1 to 15 days after the beginning of WWP2 using a porometer (AP4, Delta-T Devices, Cambridge, UK) for the WR-treated plants. g_s_ was measured at mid-morning on the two youngest completely developed leaves of five plants per cultivar and treatment. Microscopic observations were carried out on the upper and lower surfaces of the leaves. Since no stomata were found on the upper surface, all the measurements were made on the lower surface. For the control plants, measurements were made at the same time as for WR-treated plants to avoid experimental bias due to environment effect. g_s_ was thus considered as a qualitative water stress-related indicator for each cultivar independently of the others.

### Water Stress-Related Indicators

For each water stress-related indicator, measurements were carried out throughout the experiment as follows (**Figure 2**):

- the last day of WRP1 and WRP2;
- the last day of WWP1;
- 15 days (sufficient duration for recovery) after the beginning of WWP2.

For the control plants, measurements were made at the same time as WR-treated plants.

### Relative Water Content (RWC)

Two of the youngest completely developed leaves were randomly collected from three plants per cultivar and treatment. Two 1-cm-diameter foliar discs per leaf were weighed immediately after sampling to determine fresh mass. The discs were placed in distilled water and kept in the dark at 4∘C for 12 h and then re-weighed to record turgid weight. Dry mass was weighed after lyophilization of the foliar discs. Relative water content (RWC) was calculated as follows:

RWC = ((fresh weight – dry weight)/(turgid weight – dry weight)) ^∗^ 100

After collection of foliar discs, the remains were immediately frozen in liquid nitrogen and kept at -80∘C. They were then lyophilized and ground into a fine dust, which was thereafter used for analysis of hormone, sugar, and proline content.

### Hormone Content

For each sample, 10 mg of freeze-dried powder were extracted with 0.8 mL of acetone/water/acetic acid (80/19/1 v:v:v). ABA, SA, JA, and indole-3-acetic acid (IAA) stable labeled isotopes used as internal standards were prepared as described in Le Roux et al. (2014). Two ng of each standard were added to the sample. For CK, 10 stable labeled isotopes were used as internal standards and added as follows: 1 ng of ^2^H_5_-t-Z7G (*trans*-zeatin-7-glucoside), 1 ng of ^2^H_5_-t-Z9G (*trans*-zeatin-9-glucoside), 1 ng of ^2^H_5_-t-ZOG (*trans*-zeatin *O*-glucoside), 1 ng of ^15^N-t-Z (*trans*-zeatin), 1 ng of ^2^H_5_-t-ZROG (*trans*-zeatin riboside *O*-glucoside), 1 ng of ^2^H_5_-t-ZR (*trans*-zeatin riboside), 1 ng of ^2^H_6_-iPRMP (isopentenyl adenosine monophosphate), 1 ng of ^2^H_6_-iP (isopentenyl adenine), 1 ng of ^2^H_5_-t-ZRMP (*trans*-zeatin riboside monophosphate), 0.1 ng of ^15^N-iPR (isopentenyl adenosine). The extract was vigorously shaken for 1 min, sonicated for 1 min at 25 Hz, shaken for 10 min at 4∘C in a Thermomixer (Eppendorf^®^), and then centrifuged (8,000 *g*, 4∘C, 10 min). The supernatants were collected and the pellets were re-extracted twice with 0.4 mL of the same extraction solution, then vigorously shaken (1 min) and sonicated (1 min; 25 Hz). After the centrifugations, the three supernatants were pooled and dried (final volume: 1.6 mL).

Each dry extract was dissolved in 140 μL of acetonitrile/water (50/50 v/v), filtered and analyzed using a Waters Acquity Ultra-Performance Liquid Chromatograph coupled to a Waters Xevo Triple quadrupole mass spectrometer (UPLC-ESI-MS/MS). The compounds were separated on a reverse-phase column (Uptisphere C18 UP3HDO, 100^∗^2.1 mm^∗^3 μm particle size; Interchim, France) using a flow rate of 0.4 mL min^-1^ and a binary gradient: (A) 0.1% acetic acid in water (v/v); and (B) acetonitrile with 0.1% acetic acid. For CK, the solvent gradient was applied as follows: (time, % A): (0 min., 95%), (12 min., 40%), (13 min., 0%), (16 min., 95%); and the column temperature was 40∘C. For ABA, SA, JA and IAA, we used the following binary gradient (time, % A): (0 min., 98%), (3 min., 70%), (7.5 min., 50%), (8.5 min., 5%), (9.6 min., 0%), (13.2 min., 98%), (15.7 min., 98%). Mass spectrometry was conducted in electrospray and Multiple Reaction Monitoring scanning mode (MRM mode), in positive ion mode for IAA, and in negative ion mode for the other hormones. Relevant instrumental parameters were set as follows: capillary 1.5 kV (negative mode), source block and desolvation gas temperatures of 130 and 500∘C, respectively. Nitrogen was used to assist the cone and desolvation (150 and 800 L h^-1^, respectively), argon was used as the collision gas at a flow rate of 0.18 mL min^-1^.

Samples were reconstituted in 140 μL of 50/50 acetonitrile/H_2_O (v/v) *per* mL of injected volume. The limit of detection (LOD) and the limit of quantification (LOQ) were extrapolated for each hormone from calibration curves and samples using the Quantify module of MassLynx software, version 4.1.

### Sugar Content

Sucrose, glucose, and fructose contents were determined by high-performance liquid chromatography (HPLC) using a Carbopac PA-1 column (Dionex Corp., Sunnyvale, CA, USA), according to Vandecasteele et al. (2011). Ten mg of powder from each sample were homogenized with 1.3 ml of 80% aqueous ethanol at 80∘C for 30 min. They were then centrifuged at 5500 rpm for 5 min at 4∘C. Supernatants were collected and the solvent was removed using a SpeedVac concentrator. Remaining pellets were re-suspended in 0.6 mL water. After appropriate dilution, extracts were analyzed along with standards (glucose: 10 mg L^-1^; fructose: 10 mg L^-1^; sucrose: 20 mg L^-1^; raffinose: 40 mg L^-1^; stachyose: 40 mg L^-1^; melizitose: 40 mg L^-1^).

### Proline Content

Proline content was determined by an acquity ultraperformance liquid chromatograph with diode array detection (UPLC-DAD system, Waters, Milford, MA, USA), according to Jubault et al. (2008). A methanol–chloroform–water-based extraction was performed on 10 mg of powder from each sample as described by Gravot et al. (2010). Each extract was dried under vacuum. Dry residues were resuspended in 50 μl of ultrapure water and 10 μl were used for the derivatization using the AccQ-Tag Ultra Derivatization Kit (Waters, Milford, MA, USA). 3-aminobutyric acid was used as the internal standard.

### Physiological Data Analysis

Data collected were divided as follows: (A) data obtained at the end of WRP1 and WRP2 that characterize plant responses to water restriction; and (B) data obtained during WWP1 and WWP2 at least 15 days after the beginning of the re-watering, which characterize the recovery capacity of the plant. Each group of data was analyzed separately along with its corresponding control data. When using a large number of variables it is often not clear which ones are relevant and/or redundant to describe the observed variability. Therefore, data were first subjected to a principal component analysis (PCA) to select variables that (i) best explain the physiological variability and (ii) are the least correlated among themselves (Ky et al., 2013; Granda et al., 2014; Takahashi et al., 2014). PCA is a well-established dimensionality reduction method (Eklöv et al., 1999; Pagola et al., 2009; Verbanck et al., 2013) that makes it possible to describe a multidimensional dataset using a small number of variables while retaining as much information as possible. The reduction is achieved by transforming the original variables into a new set of independent continuous variables, referred to as the principal components (PCs).

The selected variables were subjected to a Kruskal–Wallis test to evaluate the genotype and watering effects and to a Mann–Whitney–Wilcoxon test for comparisons of means. Differences with *p* < 0.05 were considered to be significant. Data analyses were carried out using R 3.0.2 software.

## Results

### Genotype and Watering Effects and their Interaction on Plant Architecture

Genotype and watering effects as well as their interaction (ANOVA) were significant for all the architectural variables measured.

For the watering effect, a significantly lower effect was revealed for WR-treated plants compared to control plants for all the variables (**Table 1**), with a difference of -34.2% for NbDetA, -19.8% for L_LA, -15.5% for AngLA/Cord, -6.7% for NbLA, -6.3% for NbBrO, and -5.2% for NbMet_LA.

**Table 1:** Mean of six architectural variables measured on control plants and plants subjected to a water restriction (WR) treatment of eight rose genotypes.

Genotypes	NbDetA				L_LA (cm)				AngLA/Cord (°)			
	Control	WR	Mean	Variation (%)	Control	WR	Mean	Variation (%)	Control	WR	Mean	Variation (%)
‘Baipome’	337.7	210.0	273.9 F	-37.8	23.5	18.3	20.9 CD	-22.1	99.2	79.3	89.2 E	-20.1
‘Blush Noisette’	68.9	72.6	70.8 C	5.3	28.9	15.1	22.0 D	-47.8	86.4	66.2	76.3 C	-23.4
‘The Fairy’	242.7	94.1	168.4 E	-61.2	21.8	18.2	20.0 BC	-16.5	98.6	69.8	84.2 D	-29.2
‘Old Blush’	54.7	42.2	48.5 B	-22.9	23.6	20.2	21.9 D	-14.4	74.6	71.5	73.1 BC	-4.2
‘Perle d’Or’	24.8	39.2	32.0 A	58.1	20.9	19.0	20.0 BC	-9.1	71.9	64.4	68.1 B	-10.4
Hw20	106.0	39.7	72.9 C	-62.6	19.1	17.8	18.4 B	-6.8	78.3	61.9	70.1 B	-20.9
Hw336	154.9	149.2	152.1 D	-3.7	20.0	17.6	18.7 B	-12.0	82.2	83.2	82.7 D	1.2
Hw154	23.0	19.0	21.0 A	-17.4	15.9	12.6	14.3 A	-20.8	54.5	49.5	52.0 A	-9.2
**Mean**	126.6 a	83.3 b		-34.2	21.7 a	17.4 b		-19.8	80.7 a	68.2 b		-15.5

**Genotypes**	**NbLA**				**NbBrO**				**NbMet_LA**			
	**Control**	**WR**	**Mean**	**Variation (%)**	**Control**	**WR**	**Mean**	**Variation (%)**	**Control**	**WR**	**Mean**	**Variation (%)**

‘Baipome’	52.8	55.9	54.4 E	5.9	5.5	4.9	5.2 C	-10.9	14.8	13.5	14.2 E	-8.8
‘Blush Noisette’	27.6	46.6	37.1 D	68.8	4.7	4.6	4.7 B	-2.1	11.0	8.7	9.9 A	-20.9
‘The Fairy’	41.4	24.7	33.1 C	-40.3	4.8	4.2	4.5 AB	-12.5	13.2	13.0	13.1 D	-1.5
‘Old Blush’	37.0	29.9	33.5 C	-19.2	4.4	4.1	4.3 A	-6.8	9.5	10.5	10.0 A	10.5
‘Perle d’Or’	11.9	13.7	12.8 A	15.1	4.2	4.2	4.2 A	0.0	11.0	10.6	10.8 B	-3.6
Hw20	30.4	16.8	23.6 B	-44.7	5.2	4.4	4.8 B	-15.4	11.4	10.1	10.7 B	-11.4
Hw336	36.1	33.9	35.0 CD	-6.1	5.2	5.1	5.2 C	-1.9	11.9	11.4	11.6 C	-4.2
Hw154	14.1	13.2	13.7 A	-6.4	4.2	4.1	4.2 A	-2.4	10	9.5	9.7 A	-5.0
**Mean**	31.4 a	29.3 b		-6.7	4.8 a	4.5 b		-6.3	11.6 a	11 b		-5.2

*Mean followed by the same lowercase letter within the same line are not significantly different (Newman–Keuls test, *p* < 0.05). Mean followed by the same uppercase letter within the same column are not significantly different (Newman–Keuls test, *p* < 0.05). Mean of 10 plants per genotype and watering treatment are shown. Variations are calculated between the two watering treatments with respect to control*.

For the genotype effect, the analysis revealed different groupings of genotypes depending on the variables (**Table 1**). For NbDetA, six groups were defined, with Hw154 and ‘Perle d’Or’ (group A) characterized by the lowest numbers (21.0 and 32.0 respectively) and ‘Baipome’ (group F) with the highest number of axes (273.9). For L_LA, four groups were found, with Hw154 (group A) characterized by the shortest long axes (14.3 cm) and ‘Old Blush’ and ‘Blush Noisette’ (group D) with the greatest length (21.9 and 22.0 cm respectively). For AngLA/Cord, five groups were determined, with Hw154 (group A) characterized by the smallest angle (52.0∘) and ‘Baipome’ (group E) with the widest angle (89.2∘). For NbLA, five groups were identified, with ‘Perle d’Or’ and Hw154 (group A) characterized by the lowest number of long axes (12.8 and 13.7 respectively) and ‘Baipome’ (group E) with the highest number (54.4). For NbBrO, three groups were distinguished, with Hw154, ‘Perle d’Or,’ and ‘Old Blush’ (group A) characterized by the lowest order number (4.2, 4.2, and 4.3, respectively) and ‘Baipome’ and Hw336 (group C) by the highest order number (5.2). For NbMet_LA, five groups were established, with Hw154, ‘Blush Noisette,’ and ‘Old Blush’ (group A) characterized by the lowest number of metamers on long axes (9.7, 9.9, and 10.0 respectively) and ‘Baipome’ (group E) with the highest number (14.2).

**FIGURE 3:**
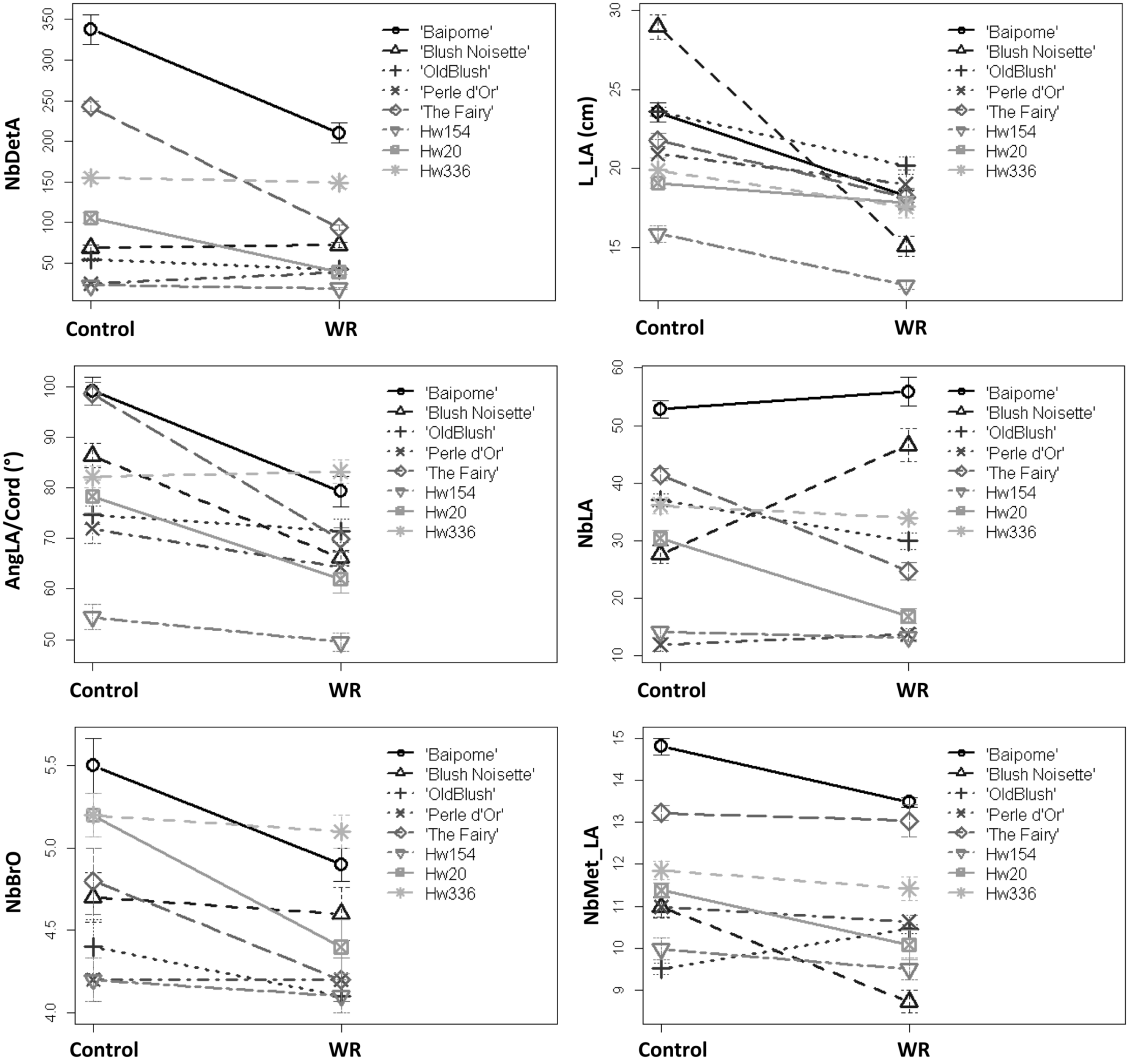
**Mean of six architectural variables measured on control plants and plants subjected to a water restriction (WR) treatment of eight rose genotypes**. Mean and standard error (SE) of 10 plants per genotype and treatment are shown.

There was a strong interaction between genotype and watering for all the variables measured (**Figure 3**). The interactions were due to different genotype response amplitudes for all variables and even significant opposite responses to WR treatment for NbDetA, NbMet_LA, and NbLA. The extreme amplitudes were (for WR-treated plants compared to control plants): for NbDetA, -62.6% (Hw20) and +58.1% (‘Perle d’Or’); for L_LA, -47.8% (‘BlushNoisette’) and -6.8% (Hw20); for AngLA/Cord, -29.2% (‘The Fairy’) and +1.2% (Hw336); for NbLA, -44.7% (Hw20) and +68.8% (‘Blush Noisette’); for NbBrO, -15.4% (Hw20) and 0.0% (‘Perle d’Or’); for NbMet_LA, -20.9% (‘BlushNoisette’) and +10.5% (‘Old Blush’).

**FIGURE 4:**
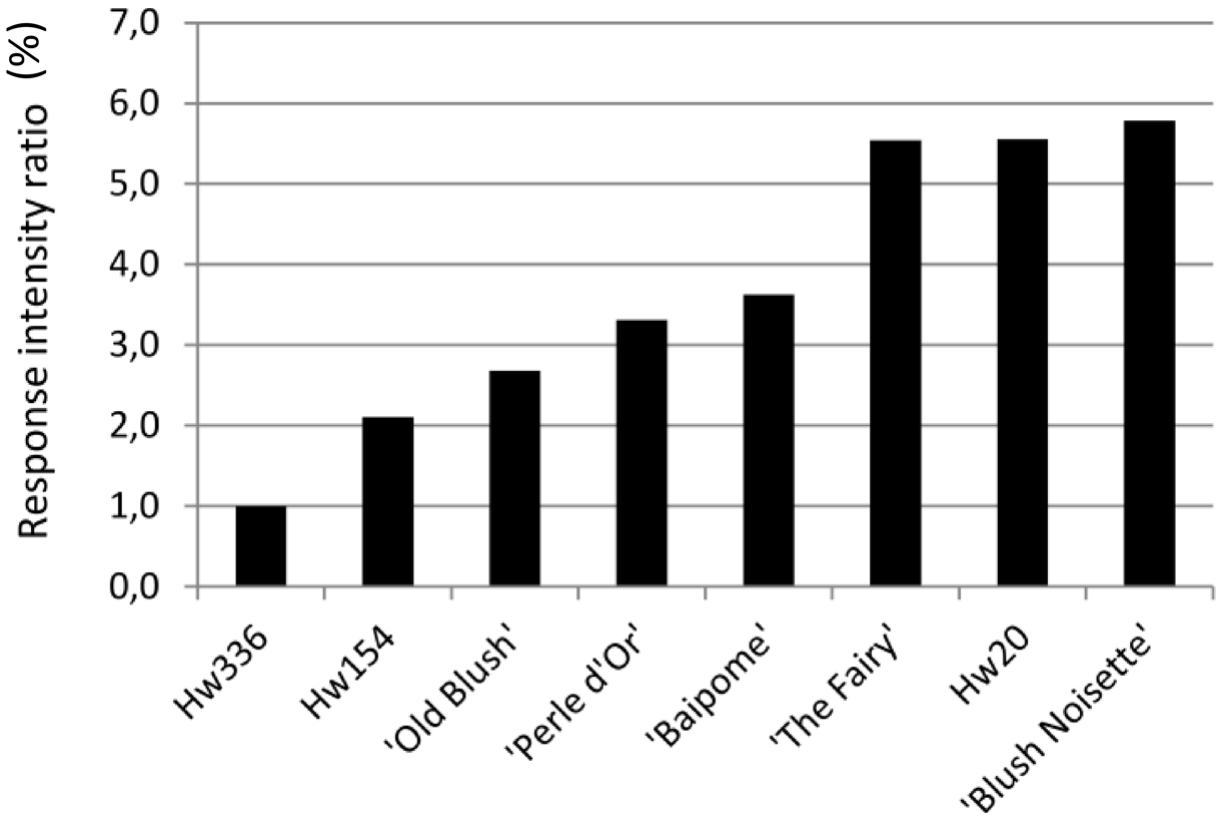
**Intensity of the architectural response per genotype**.

The intensity of the architectural response to water restriction of each genotype allowed us to distinguish three groups (**Figure 4**). Compared to Hw336 that only slightly responded, Hw154, ‘Old Blush,’ ‘Perle d’Or,’ and ‘Baipome’ had moderate responses (2 to 4-fold), and ‘The Fairy,’ Hw20, and ‘Blush Noisette’ had the strongest responses (5 to 6-fold).

### Assessment of Plant Transpiration by Stomatal Conductance

**FIGURE 5:**
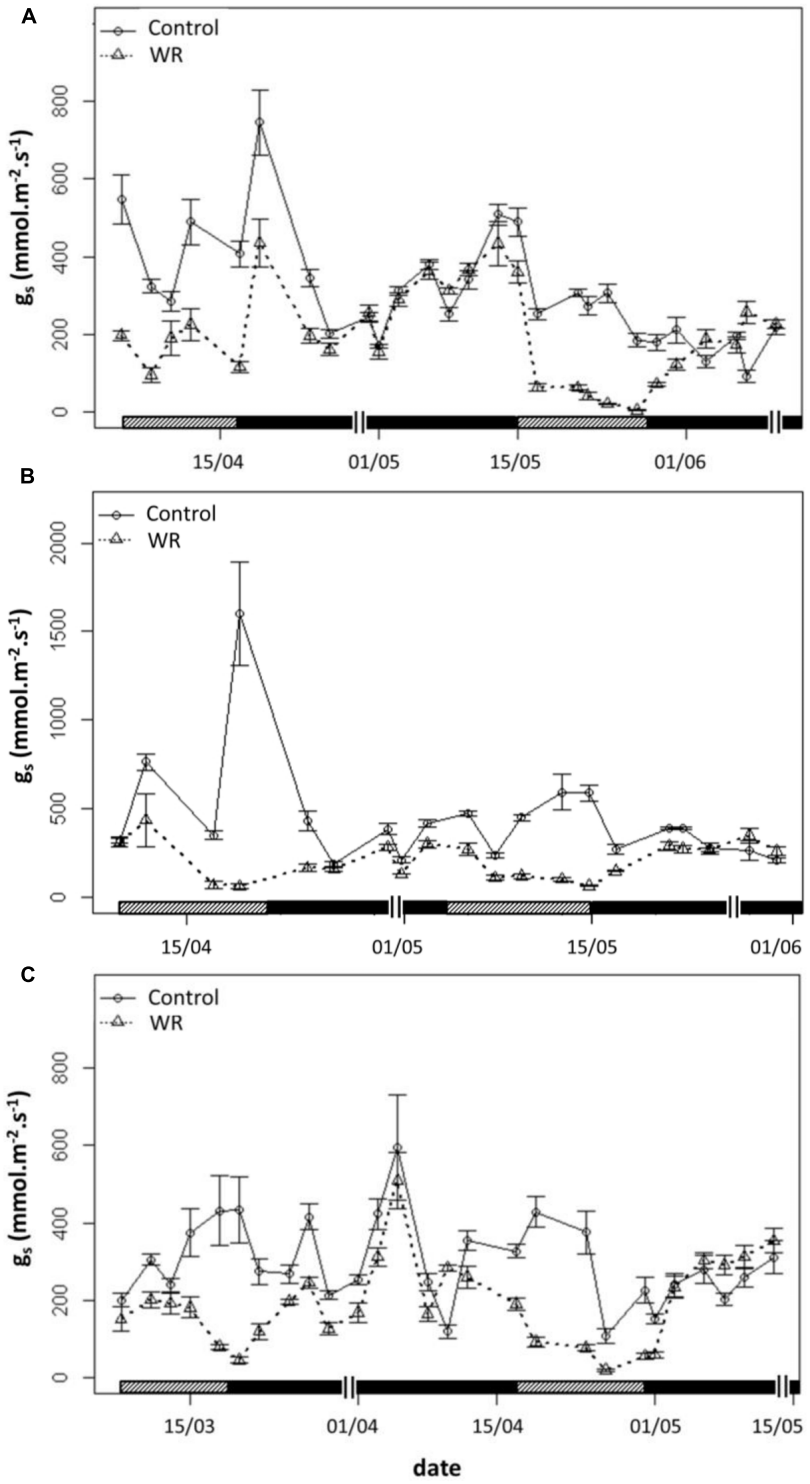
**Stomatal conductance (g_s_) during water restriction (WRP) and re-watering (WWP) periods**. All measurements were carried out on the two youngest leaves completely developed of control plants 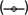 and plants subjected to a WR treatment 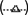 for the three genotypes ‘Baipome’ **(A)**. ‘The Fairy’ **(B)**, and Hw336 **(C)**. Hatched bars indicate WRP1 and WRP2 periods and closed bars indicate WWP1 and WWP2 periods. A double line (||) delimits the 15-days period after each re-watering. Mean and SE of five plants per genotype and treatment are shown.

For each genotype, g_s_ declined progressively as water restriction duration persisted whether in WRP1 or WRP2, compared to control plants. After 15 days of re-watering, g_s_ gradually went back up to the control values (**Figure 5**).

### Selecting the Most Relevant Water Stress-Related Variables

**FIGURE 6:**
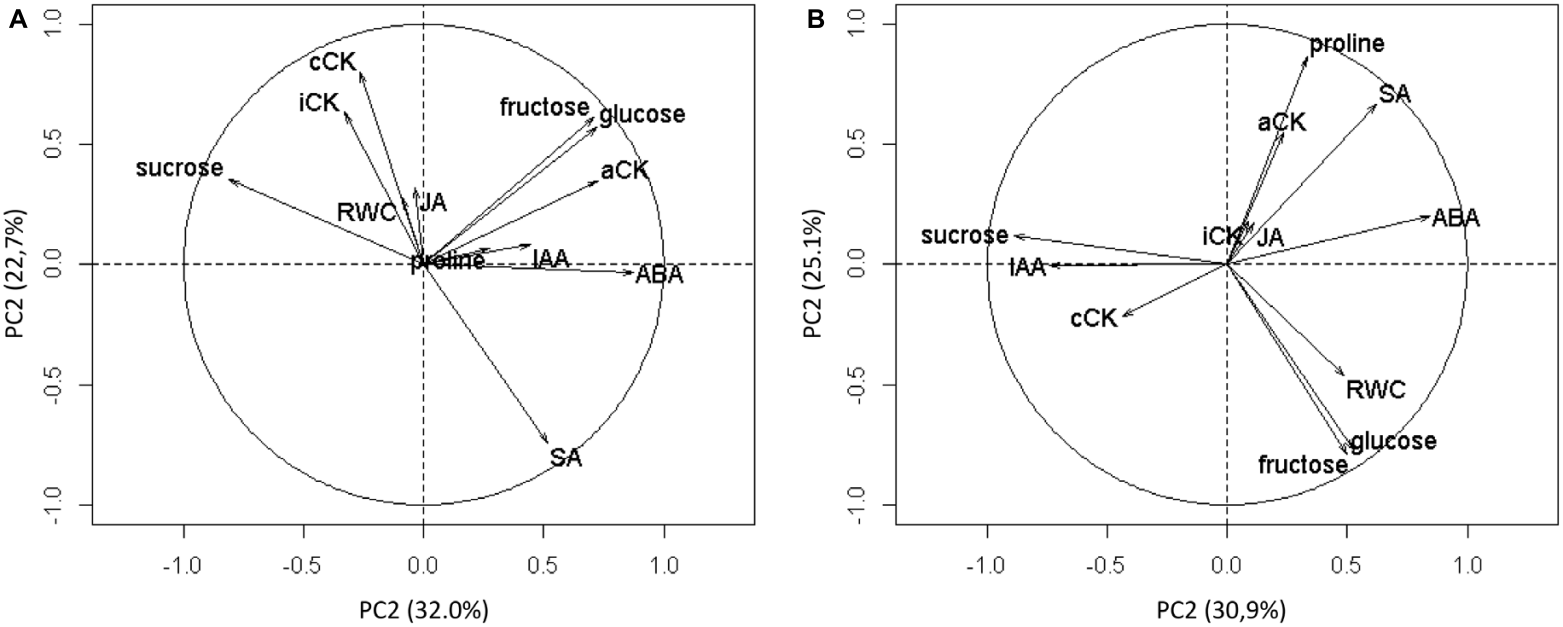
**Circles of correlations of the principal components 1 (PC1) and 2 (PC2) of the principal component analysis (PCA) built using 12 physiological variables**. The variables were measured during WRP1 and WRP2 periods **(A)** and during WWP1 and WWP2 periods **(B)**.

Twelve water stress-related variables were measured at the end of each WRP (dataset A) and at least 15 days after the beginning of each WWP (dataset B) for control plants and WR-treated plants. They were then subjected to a PCA (**Figure 6**). For the analysis of dataset A, the first two PCs accounted for 32.0 and 22.7%, respectively, or 54.7% of the total variability. For the analysis of dataset B, the first two PCs accounted for 30.9 and 25.1%, or 56.0% of the total variability.

For each of the two analyses, relevant variables were selected based on one criterion: to contribute the most to the major component formation.

For the analysis of dataset A, ABA (19.6%) and sucrose (17.1%) were selected for PC1, and conjugated cytokinin (cCK) (21.4%), SA (18.3%), and intermediate cytokinin (iCK) (18.3%) were selected for PC2 (**Table 2**). For the analysis of dataset B, ABA (20.2%) and sucrose (22.2%) were chosen for PC1, and proline (25.5%), glucose (19.2%), and fructose (18.7%) were chosen for PC2. Since glucose and fructose were highly correlated (*r* = 0.99) and thus gave redundant information, fructose was arbitrarily selected.

**Table 2:** Contribution of the 12 physiological variables to the formation of principal components (PC) 1 and 2 of the PCA of the datasets A **(A)** and B **(B)**.

Variables	Contribution of variables (%)	
	PC1	PC2
**(A)**
aCK	13.1	6.2
**iCK**	2.0	**18.2**
**cCK**	2.9	**21.4**
**Sucrose**	**17.1**	4.4
Glucose	14.0	10.4
Fructose	13.5	12.1
**ABA**	**19.6**	0.0
**SA**	7.7	**18.3**
IAA	7.1	0.4
JA	0.1	2.5
RWC	0.8	5.1
Proline	2.3	1.0
**(B)**
aCK	2.1	9.1
iCK	0.1	3.6
cCK	6.2	0.5
**Sucrose**	**22.2**	0.1
Glucose	7.7	19.2
**Fructose**	8.5	**18.7**
**ABA**	**20.2**	0.5
SA	11.5	12.3
IAA	13.3	0.0
JA	0.1	3.2
RWC	4.4	7.4
**Proline**	3.7	**25.5**

Selected variables are in bold.

Therefore, the most explanatory variables of the observed variability for water restriction were ABA, sucrose, cCK, SA and iCK, and the ones best explaining the observed variability for re-watering were ABA, sucrose, fructose, and proline.

The Kruskal–Wallis test showed no significant difference either between the two WRP or between the two WWP for these variables. Thus, data obtained within each group were pooled for further analyses.

### Genotype and Watering Effect on Selected Water Stress-Related Variables for WRP

A significant watering effect was shown for ABA content, with an increase in plants subjected to water restriction compared to control plants (+118.3%; **Table 3**). A significant increase of +154.5, +115.0, and +106.7% for ‘Baipome,’ Hw336, and ‘The Fairy,’ respectively, was observed. However, no significant genotype effect was revealed for ABA content. Although standard deviations were high, a significant watering effect was shown for sucrose content, with a decrease in plants subjected to water restriction compared to control plants (-68.6%; **Table 3**). A significant decrease of -79.9, -70.8, and -65.2% for Hw336, ‘The Fairy’ and ‘Baipome,’ respectively, was observed. No significant genotype effect was shown for sucrose contents.

**Table 3:** Abscisic acid (ABA), salicylic acid (SA), sucrose, conjugated cytokinins (cCK), and intermediate cytokinins (iCK) mean contents of three genotypes (‘Baipome,’ ‘The Fairy,’ and Hw336) determined in control plants and treated-plants during water restriction periods (WRPs).

Genotypes	ABA (ng/mg DW)			SA (ng/mg DW)			Sucrose (mg/L)			cCK (ng/g DW)			iCK (ng/g DW)		
	Control	WRP	Mean	Control	WRP	Mean	Control	WRP	Mean	Control	WRP	Mean	Control	WRP	Mean
‘Baipome’	4.4 a	11.2 b	8.1 A	6.4 a	12.9 a	9.9 A	494.4 a	171.9 b	318.5 A	19.7 a	25.1 a	22.6 A	31.9 a	24.9 a	28.1 A
‘The Fairy’	7.5 a	15.5 b	11.1 A	27.8 a	41.2 a	33.9 B	325.7 a	95.0 b	220.8 A	15.7 a	4.7 b	10.7 B	19.3 a	19.9 a	19.6 A
Hw336	6.0 a	12.9 b	9.4 A	26.6 a	34.5 a	30.6 B	210.1 a	42.3 b	126.3 A	8.2 a	6.3 a	7.2 B	24.1 a	19.0 a	21.7 A
**Mean**	6.0 a	13.1 b		20.7 a	28.5 a		342.4 a	107.4 b		14.6 a	12.8 a		24.7 a	21.5 a	

*Mean followed by the same lowercase letter within the same line are not significantly different (Mann-Whitney-Wilcoxon test, *p* < 0.05). Mean followed by the same uppercase letter within the same column are not significantly different (Kruskal–Wallis test, *p* < 0.05). Mean of three plants per genotype and watering treatment are shown*.

*DW, dry weight*.

No significant watering effect was shown for SA content. However, a significant genotype effect was revealed, with a SA content three times higher for ‘The Fairy’ and Hw336 compared to ‘Baipome.’ Although no significant watering effect was shown for cCK content, a significant decrease was observed only for ‘The Fairy’ (-70.1%). A genotype effect was revealed, with a cCK content 2–3 times higher for ‘Baipome’ compared to ‘The Fairy’ and Hw336.

No significant watering and genotype effect was shown for iCK content.

### Genotype and Watering Effect on Selected Water Stress-Related Variables for WWP

No significant watering effect was shown for any of the selected variables (**Table 4**). However, a significant genotype effect was revealed for ABA and proline contents. ABA content was two times higher in ’The Fairy’ than in ‘Baipome’ and Hw336, and proline content was five times higher in ‘The Fairy’ and Hw336 than in ‘Baipome.’

**Table 4:** Abscisic acid (ABA), sucrose, fructose, and proline mean contents of three genotypes (‘Baipome,’ ‘The Fairy,’ and Hw336) determined in control plants and treated-plants during well-watered periods (WWPs).

Genotypes	ABA (ng/mg DW)			Sucrose (mg/L)			Fructose (mg/L)			Proline (nmol/mg)		
	Control	WWP	Mean	Control	WWP	Mean	Control	WWP	Mean	Control	WWP	Mean
‘Baipome’	4.7 a	6.1 a	5.4 A	278.2 a	271.1 a	274.7 A	408.5 a	280.4 a	344.5 A	1.4 a	1.3 a	1.3 A
‘The Fairy’	11.7 a	9.9 a	10.8 B	60.7 a	136.2 a	98.4 A	401.2 a	203.0 a	302.1 A	7.5 a	5.6 a	6.6 B
Hw336	6.5 a	4.9 a	5.7 A	212.3 a	384.8 a	298.5 A	197.7 a	155.2 a	176.5 A	5.7 a	7.4 a	6.6 B
**Mean**	7.4 a	6.8 a		191.7 a	271.6 a		273.0 a	623.8 a		4.7 a	4.7 a

*Mean followed by the same lowercase letter within the same line are not significantly different (Mann–Whitney–Wilcoxon test, *p* < 0.05). Mean followed by the same uppercase letter within the same column are not significantly different (Kruskal–Wallis test, *p* < 0.05). Mean of three plants per genotypes and watering treatment are shown*.

*DW, dry weight*.

## Discussion

We showed that the application of the alternation of restriction and re-watering periods led to a significant decrease for all of the architectural variables measured, indicating a strong impact of water supply on the control of plant architecture. This effect was particularly pronounced for L_LA (-19.8%). Similar observations were reported for *R. hybrida* ‘Knock-Out,’ with a decrease in the length of order 1 axes of -13% for a WRP of 21 days, and -15% for a period of 35 days; the water potential of the substrate was -26 kPa (Demotes-Mainard et al., 2013). Similar results were obtained for other species, such as *C. roseus* (Jaleel et al., 2008), *C. coggygria,* or *Forsythia* x *intermedia* (Cameron et al., 2006). The decrease of the length of long axes was mainly due to the decrease in the length of their own metamers since the watering effect on NbMet_LA was weak; the difference was less than one metamer between watering treatments. Similarly, Crespel et al. (2014) showed that NbMet_LA was stable when subjecting the same eight genotypes to different levels of cumulated radiation over 2 years. Likewise, the application of water restriction on *C. coggygria* and *Forsythia* x *intermedia* led to a significant decrease in the length of the metamers but had no effect on their number (Cameron et al., 2008).

Strong decrease in the number of axes (-34.2%) was also found following the application of water restriction. The same decrease was observed after the application of a 10-weeks water restriction (irrigation at 50% of evapotranspiration potential) in *Cornus alba* and *Lonicera periclymenum* (Cameron et al., 2006). Watering effect on NbLA was significant but weak (-6.7%). Morel et al. (2009) showed that the number of long axes decreases with the number of branching orders. Consequently, relatively few long axes were exposed to water restriction which was applied after budbreak on order 2 axes, i.e., at the time of formation of higher order axes. In our study, significant decreases in the number of determined axes were mainly observed for orders 3 and 4 (Supplementary Table S1), as reported in *R. hybrida* ‘Radrazz’ submitted to drought/re-watering cycles (Huché-Thélier et al., 2013).

The insertion angle of the cord of long axes decreased by -15.5%, revealed by the more upright bearing of plants subjected to WR than that of the control plants. This difference could be explained by the lower biomass load (stems and leaves) borne by the long axes, as was already observed in rose (Crespel et al., 2014) and in apricot (Alméras et al., 2004).

In general, the eight genotypes responded similarly to WR, with a decrease in the values of the different architectural variables. Nevertheless, a significant genotype × watering interaction was observed for the six architectural variables and was in large part due to the amplitudes of the different responses between genotypes.

The intensities of the architectural response made it possible to distinguish three groups: Hw336, which had a very low response to WR; Hw154, ‘Old Blush,’ ‘Perle d’Or,’ and ‘Baipome,’ which had a moderate response; and ‘The Fairy,’ Hw20, and ‘Blush Noisette,’ which had a very strong response. Similarly, Crespel et al. (2014) showed that ‘The Fairy’ was one of the genotypes that responded the most to the quantity of radiation perceived by the plant, that ‘Baipome’ responded moderately, and that Hw336 responded very weakly, particularly for the variables AngLA/Cord and NbBrO. The weak response observed for Hw336 could be explained by its strong heterozygotic nature since Hw336 is an interspecific hybrid resulting from a cross between a dihaploid of cultivated rose and a hybrid of *R. wichurana* (Gillespie and Turelli, 1989; Scheiner, 1993). ‘Baipome’ is the product of a genetic breeding program to improve water deficit tolerance (Crespel, personal communication), which would explain its more moderate response compared to that of ‘The Fairy.’ A difference in the intensity of responses to a water deficit between four species (*R. hybrida* ‘Dr. Huey,’ *R.* x *fortuniana*, *R. multiflora,* and *R. odorata*) of the genus *Rosa* was recently reported (Niu and Rodriguez, 2009). However, this effect was assessed on plant growth using only four biomass variables and no architectural variables.

In order to explain the three different intensities of architectural responses between genotypes revealed by our study, a physiological analysis was carried out on ‘The Fairy,’ ‘Baipome,’ and Hw336, arbitrarily selected and representing each of the groups.

Differences were observed between water treatments for WRP and could be mainly explained by ABA and sucrose that contributed the most to the formation of PC1 of the PCA (32.0%). The concentration in ABA in the leaf doubled after the application of water resttriction, which is in consistence with the findings of Giday et al. (2014) in potted roses and in other species such as *Mangifera indica* (Zaharah and Razi, 2009), *E. globulus* (Granda et al., 2014), and *Vitis vinifera* (Stoll et al., 2000). Stomatal closure due to the accumulation of ABA in the leaf is one of the first responses of the plant to a water deficit (Jones and Mansfield, 1970; Wilkinson and Davies, 2002; Chaves et al., 2003; Tallman, 2004) and could explain the decrease in g_s_ observed in our study. A significant drop in sucrose content (-68.6%) was also observed in the leaves. It could result from: (i) the decrease in the photosynthetic rate in relation to the drop in g_s_ (Chaves, 1991; Lawlor and Cornic, 2002); (ii) the stimulation of sucrose breakdown in the leaf under water deficit conditions (Keller and Ludlow, 1993; Trouverie et al., 2003) leading to increased hexoses content (Supplementary Table S2). Glucose and fructose play the role of osmoregulators (Turner, 1996) and precursors for the biosynthesis of osmoprotectors like proline and polyamines (Szepesi et al., 2005, 2009), required for the tolerance of the plant to water deficit.

Increase of ABA and decrease of sucrose could explain, at least in part, (i) the decrease in the number of bursting buds and therefore, the number of axes and (ii) the decrease in length of long axes and therefore, of metamers. Indeed, budbreak and shoot growth could be inhibited by an increase in ABA (Cline and Oh, 2006; Sreenivasulu et al., 2012; Reddy et al., 2013; Leduc et al., 2014) and a decrease in sucrose, since sucrose was shown to stimulate budbreak in rose (Henry et al., 2011; Rabot et al., 2012; Barbier et al., 2015) and cell elongation (Wang and Ruan, 2013). Besides sucrose, cell elongation could have been limited by the decrease of turgor pressure due to water restriction (Urban, 1997).

No significant difference was observed between the water treatments for WWP, suggesting that such responses to water restriction are reversible, as was shown in *Eucalyptus pauciflora* (Kirschbaum, 1988), *Quercus pubescens* (Gallé et al., 2007), and *Populus* x *canadensis* (Marron et al., 2003).

Differences were observed between genotypes for WRP and were mainly explained by the SA and the cCK contents that contributed the most to the formation of PC2 of the PCA (25.1%). The SA content was therefore significantly greater (threefold) for ‘The Fairy’ and Hw336 compared to ‘Baipome.’ Fariduddin et al. (2003) showed that the photosynthetic rate had decreased after spraying highly concentrated SA (10^-3^ M) on the foliage of *Brassica juncea*, whereas it was higher for plants sprayed with low concentrations of SA (10^-5^ M). The photosynthetic rate of ‘Baipome’ could be thus greater than that of ‘The Fairy’ and Hw336. The cCK content in the leaves did not significantly vary between the two water treatments for any of the genotypes. Nevertheless, a significant difference was observed between genotypes: it was significantly lower (2–3 fold) for ‘The Fairy’ and Hw336 compared to ‘Baipome.’ The CKs are implicated in the growth and branching processes of the plant (Werner et al., 2001; Sakakibara, 2006). They are particularly recognized for their role in budbreak stimulation (Dieleman et al., 1997; Nordström et al., 2004; Shimizu-Sato et al., 2009). The higher concentration in cCK in ‘Baipome’ could therefore explain the greater number of axes formed. Moreover, many studies have shown the implication of CKs in the regulation of plant responses to abiotic stresses such as water stress (Pospíšilová et al., 2000).

In our study, three different CK content tendencies were observed after application of water restriction.

For ‘The Fairy,’ a significant decrease in the cCK content (-70.1%) was observed. This strong decrease associated with the increase in the ABA content (ABA/cCK = 3.32) could explain the strong decrease in the number of axes observed (-61.2%). For ‘Baipome,’ a non-significant increase in the cCK content (27.4%) was observed. The ABA/cCK ratio (0.44), lower than that of ‘The Fairy,’ could explain the more moderate decrease in the number of axes determined (-37.8%). Moreover, studies have shown that the application of CKs on the plant could stimulate stomatal opening (Ha et al., 2012); reduce the negative effects of water deficit on chlorophyll content and on the rate of photosynthesis (Metwally et al., 1998) and promote the re-establishment of stomatal conductance and the photosynthesis rate upon the return of water comfort (Rulcová and Pospíšilová, 2001). This would suggest that despite water restriction, the photosynthesis rate would be in part maintained by the stimulation of stomatal opening and the protection of the photosynthetic system by the CKs in ‘Baipome.’ For Hw336, the very low decrease in the number of axes (-3.7%) cannot be explained by the ABA/cCK ratio (2.0), suggesting the existence of other physiological mechanisms involved in the regulation of budbreak in this interspecific hybrid.

Differences were observed between genotypes for WWP and were mainly explained by ABA and proline since these two variables contributed the most to the formation of PC1 (30.9%) and PC2 (25.1%), respectively. The ABA content was higher for ‘The Fairy’ (twofold) compared to Hw336 and ‘Baipome,’ potentially limiting the resumption of budbreak even when water conditions are favorable. The proline content was higher for ‘The Fairy’ and Hw336 (fivefold) than for ‘Baipome.’ Experiments on engineering plants with enhanced proline accumulation showed high drought tolerance in petunia and tobacco (Yamada et al., 2005; Gubis et al., 2007). This seemed not to be the case in our study.

## Conclusion

This study showed, for the first time, the differences in genotypic responses to the alternation of WRP and WWP at the level of plant architecture in rose. A decrease in growth and branching was observed, with three groups of architectural responses of differing intensities – weak, moderate, and strong. Moderate and strong architectural responses are represented by ‘Baipome’ and ‘The Fairy’ respectively, two cultivated modern rose bushes used in gardening. Differences between these two responses could be explained by (for ‘Baipome’ compared to ‘The Fairy’): (i) the higher concentration of cCK in WRP, which could lead to budbreak and increase the rate of photosynthesis due to the stimulation of the stomatal opening and the protection of the photosynthetic system; (ii) the weaker concentration of SA in WRP, which could stimulate the rate of photosynthesis; and (iii) the weaker concentration of ABA in WWP, signifying that a resumption of budbreak would not be limited. Associated with the six architectural descriptors, ABA, SA and cCK, which explained the genotypic differences in our study, could be used as selection criteria for genetic breeding programs aimed at improving plant shape and tolerance to water deficit.

## Conflict of Interest Statement

The authors declare that the research was conducted in the absence of any commercial or financial relationships that could be construed as a potential conflict of interest.
