# Supplementary material for: Genotypic differences in architectural and physiological responses to water restriction in rose bush
Source: Front Plant Sci. 2015 May 26;6:355. doi: 10.3389/fpls.2015.00355 (PMC4443023; doi:10.3389/fpls.2015.00355)
Supplement: Supplementary file 1 [file Table_1.DOC]

**Table S1.** Means of four architectural variables measured on control plants and plants subjected to a water restriction treatment (WR) of eight rose genotypes. NbLA/BrO2: number of order 2 long axes, NbLA/BrO3: number of order 3 long axes, NbLA/BrO4: number of order 4 long axes, NbLA/BrO5: number of order 5 long axes. Means followed by the same lowercase letter within the same line are not significantly different (Mann-White-Wilcoxon test, p<0.05). Means of ten plants per genotype and watering treatment are shown.

| Genotypes | NbLA/BrO2 | | NbLA/BrO3 | | NbLA/BrO4 | | NbLA/BrO5 | |
| --- | --- | --- | --- | --- | --- | --- | --- | --- |
|  | Control | WR | Control | WR | Control | WR | Control | WR |
| 'Baipome' | 22.3 a | 26.5 b | 116.1 a | 86.9 b | 149.3 a | 75.1 b | 46.9 a | 21.5 b |
| 'Blush Noisette' | 7.6 a | 9.4 a | 30.6 a | 31.7 a | 28.1 a | 29.6 a | 2.7 a | 1.9 a |
| 'The Fairy' | 18.4 a | 18.1 a | 97.8 a | 59.5 b | 117.4 a | 16.0 b | 8.9 a | 0.5 a |
| 'Old Blush' | 11.1 a | 10.7 a | 24.6 a | 23.6 a | 16.2 a | 7.6 b | 2.8 a | 0.3 a |
| 'Perle d'Or' | 6.2 a | 6.2 a | 11.8 a | 16.3 b | 6.6 a | 15.5 b | 0.2 a | 1.2 a |
| Hw20 | 5.4 a | 5.4 a | 23.1 a | 19.1 b | 47.3 a | 13.7 b | 29.9 a | 1.5 b |
| Hw336 | 9.1 a | 8.0 a | 40.0 a | 38.2 a | 75.3 a | 76.4 a | 30.1 a | 26.4 a |
| Hw154 | 4.5 a | 6.0 b | 11.1 a | 7.8 b | 6.9 a | 5.1 a | 0.5 a | 0.1 a |
