# Supplementary material for: Genotypic differences in architectural and physiological responses to water restriction in rose bush
Source: Front Plant Sci. 2015 May 26;6:355. doi: 10.3389/fpls.2015.00355 (PMC4443023; doi:10.3389/fpls.2015.00355)
Supplement: Supplementary file 2 [file Table_2.DOC]

**Table S2.** Glucose and fructose mean contents of three genotypes ('Baipome', 'The Fairy' and Hw336) determined in control plants and treated-plants during water restriction periods (WRP). Means followed by the same lowercase letter within the same line are not significantly different (Mann-Whitney-Wilcoxon test, p<0,05). Means followed by the same uppercase letter within the same column are not significantly different (Kruskal-Wallis test, p<0,05). Means of three plants per genotypes and watering treatment are shown.

| Genotype | glucose (mg/L) | | |  | fructose (mg/L) | | |
| --- | --- | --- | --- | --- | --- | --- | --- |
|  | Control | WRP | Mean |  | Control | WRP |  |
| 'Baipome' | 395,3 a | 841,5 a | 601,8 A |  | 393,6 a | 819,3 a | 586,9 A |
| 'The Fairy' | 176,0 a | 600,6 a | 369,0 A |  | 169,5 a | 586,5 ab | 359,1 A |
| Hw336 | 323,4 a | 451 a | 383,1 A |  | 289,1 a | 418,8 b | 354,8 A |
| Mean | 290,6 a | 657,1 b |  |  | 276,9 a | 634,9 b |  |
